# Supplementary material for: Comorbidity analysis and clustering of endometriosis patients using electronic health records
Source: Cell Rep Med. 2025 Jul 31;6(8):102245. doi: 10.1016/j.xcrm.2025.102245 (PMC12432376; doi:10.1016/j.xcrm.2025.102245)
Supplement: Document S1. Figures S1–S3 and Tables S4, S9, and S10 [file mmc1.pdf]

Cell Reports Medicine, Volume *6*

Supplemental information

Comorbidity analysis and clustering

# of endometriosis patients

using electronic health records

Umair Khan, Tomiko T. Oskotsky, Bahar D. Yilmaz, Jacquelyn Roger, Ketrin Gjoni, Juan C. Irwin, Jessica Opoku-Anane, Noémie Elhadad, Linda C. Giudice, and Marina Sirota

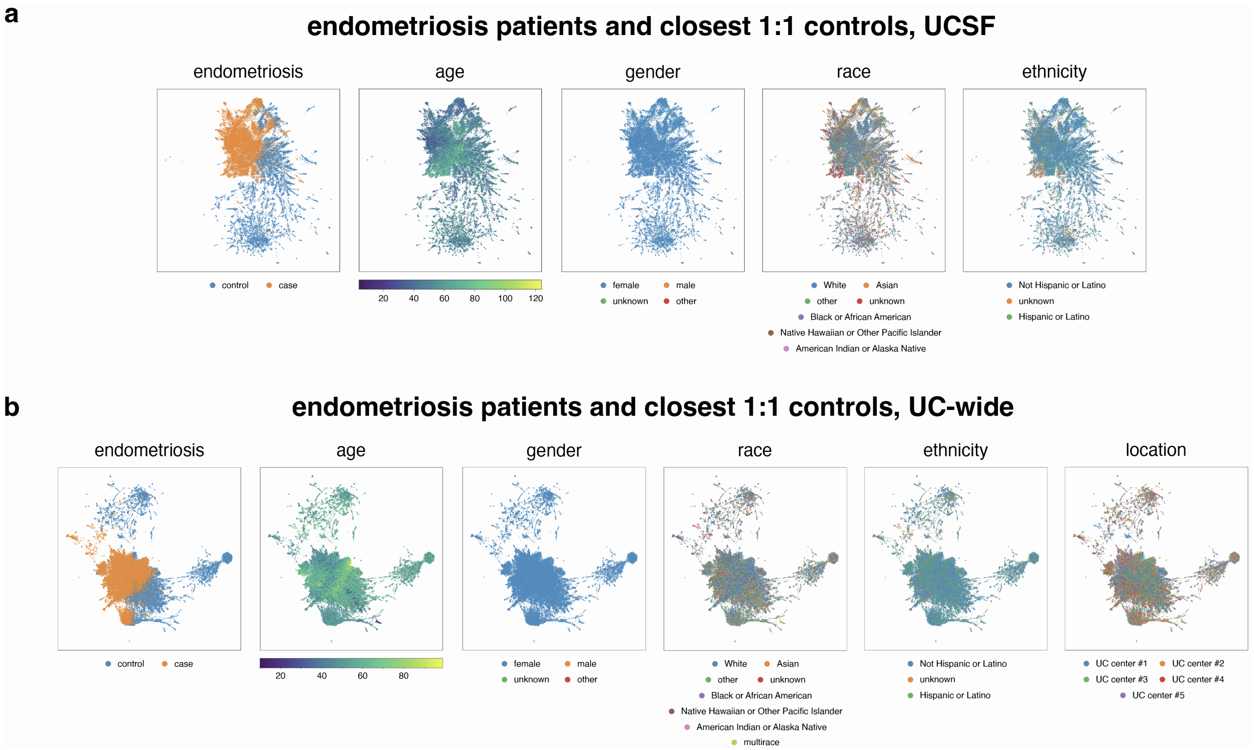

Figure S1. Two-dimensional visualization of endometriosis patients and the closest 1:1 matched controls, related to Figure 1 and STAR Methods. (a) Visualizations of UCSF patients. (b) Visualizations of UC-wide patients.

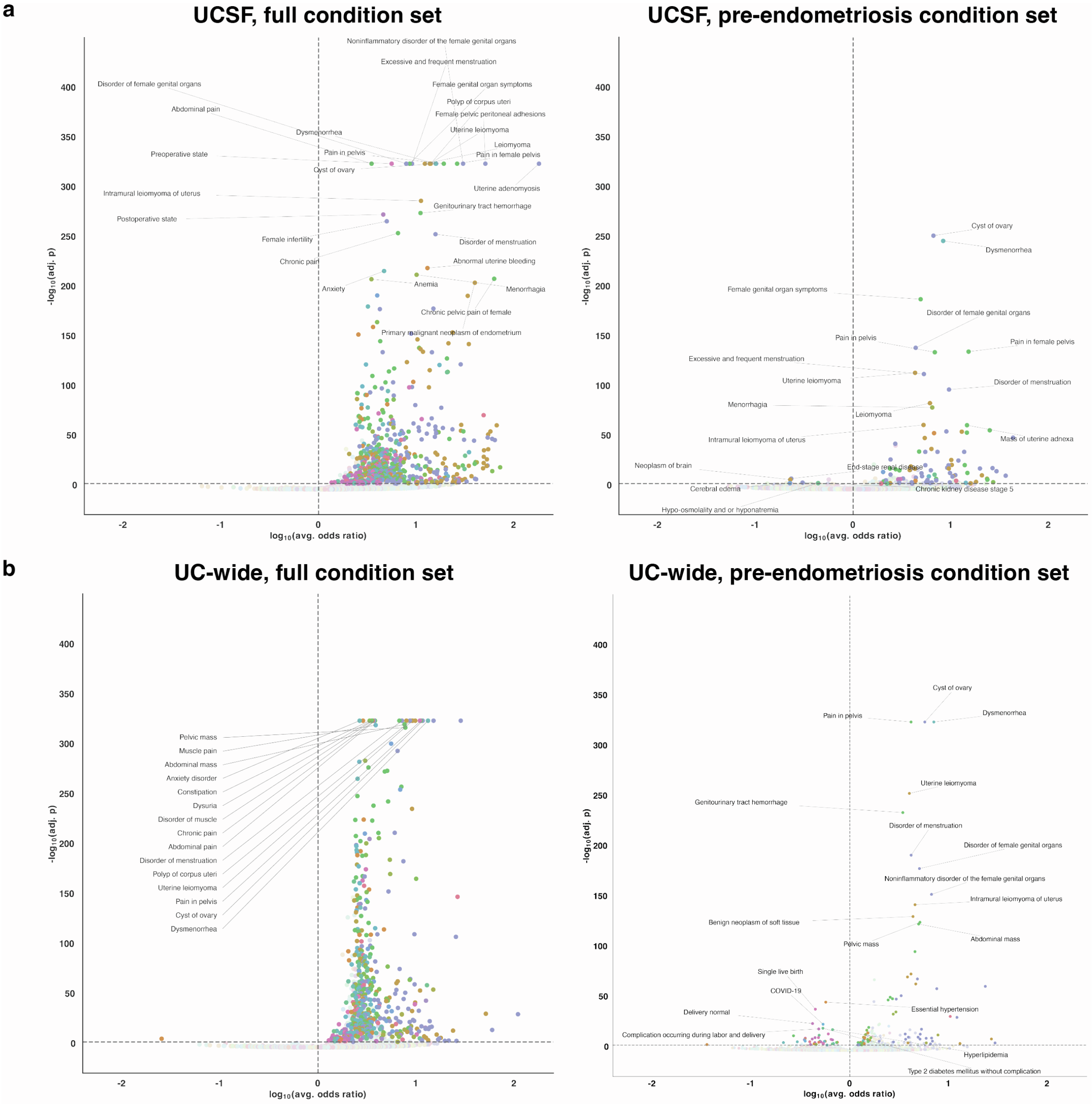

Figure S2. Volcano plots of all conditions tested for significance, related to Figure 2. (a) Visualization of UCSF condition sets. (b) Visualization of UC-wide condition sets.

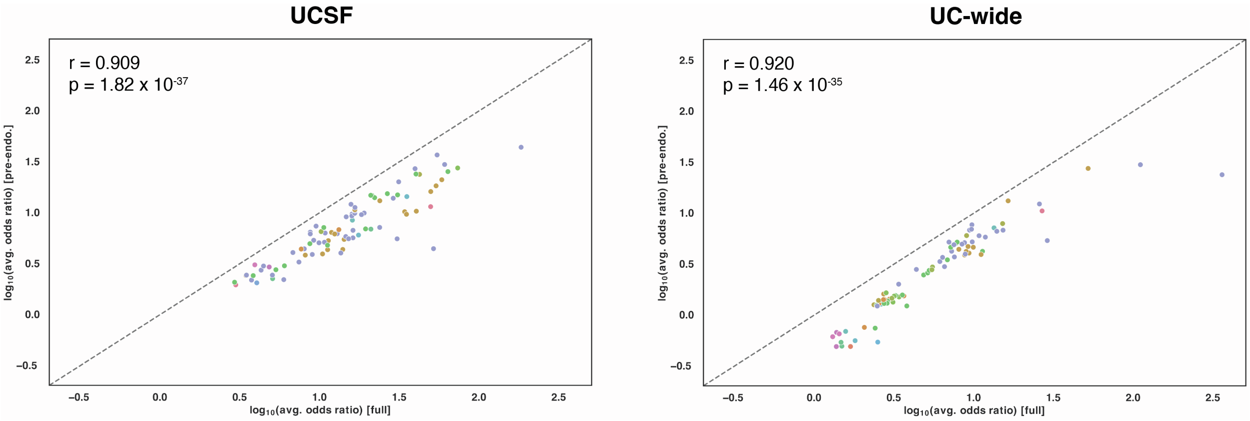

Figure S3. Concordance of odds ratios for overlapping significant associations between the analyses performed with the full set of diagnoses and only the pre-endometriosis diagnoses, both at UCSF and UC-wide, related to Figure 2.

concept name	average odds ratio (UCSF)	aggregate p-value (UCSF)	average odds ratio (UC-wide)	aggregate p-value (UC-wide)
Benign neoplasm of soft tissue	3.790	1.48E-15	4.366	4.89E-130
Benign neoplasm of uterus	15.937	2.54E-02	27.169	4.09E-08
Chronic salpingitis	11.333	6.81E-05	10.400	1.40E-30
Chronic salpingo-oophoritis	7.088	3.02E-05	12.165	1.37E-29
Corpus luteum cyst	3.974	2.82E-03	3.922	6.86E-10
Cyst of ovary	6.635	2.42E-251	5.751	0.00E+00
Disorder of female genital organs	4.366	1.49E-138	5.072	8.44E-178
Disorder of menstruation	9.578	1.39E-96	4.180	3.64E-191
Disorder of uterus	9.002	1.91E-54	6.740	4.52E-09
Dysmenorrhea	8.346	4.64E-246	7.092	0.00E+00
Dyspareunia	3.846	6.76E-16	2.951	7.46E-48
End-stage renal disease	0.232	5.22E-07	0.346	2.31E-07
Endometrial hyperplasia	9.802	1.10E-12	4.909	6.20E-10
Endometrial intraepithelial neoplasia	29.382	1.92E-14	6.873	5.66E-06
Female infertility	2.412	2.78E-26	3.313	3.27E-51
Female pelvic inflammatory disease	3.239	3.96E-07	3.674	7.66E-08
Female pelvic peritoneal adhesions	4.383	5.36E-08	5.317	7.31E-18
Genitourinary tract hemorrhage	4.736	3.26E-34	3.442	1.58E-233
Hypertrophy of uterus	7.299	6.45E-34	7.604	3.30E-58
Inflammatory disease of the uterus	13.702	6.40E-34	5.935	8.39E-09
Intramural leiomyoma of uterus	5.256	5.87E-61	4.574	8.08E-142
Left lower quadrant pain	2.976	2.64E-15	2.562	1.01E-49
Malposition of uterus	6.145	1.48E-02	5.167	6.92E-13
Noninflammatory disorder of the female genital organs	5.477	6.67E-34	6.723	4.46E-152
Noninflammatory disorder of uterus	5.486	1.74E-20	4.842	7.45E-68
Pain in pelvis	6.853	4.38E-134	4.172	0.00E+00
Pelvic mass	14.608	2.97E-53	5.135	2.63E-124
Polyp of corpus uteri	5.404	7.40E-48	4.164	8.16E-73

Premenstrual tension syndrome	2.962	8.04E-03	1.986	8.15E-06
Right lower quadrant pain	2.724	1.90E-16	2.445	5.87E-47
Submucous leiomyoma of uterus	6.330	7.25E-37	4.652	6.14E-63
Subserous leiomyoma of uterus	4.284	6.16E-17	3.837	6.71E-70
Uterine adenomyosis	43.396	3.32E-48	23.569	1.72E-60
Uterine leiomyoma	4.305	2.01E-113	4.011	1.32E-252

Table S4. Conditions significantly associated with endometriosis across both the UCSF and UCHDW datasets, using only pre-endometriosis diagnoses, related to Figure 2.

concept ID	concept name
139882	Endometriosis in scar of skin
194420	Endometriosis of fallopian tube
194421	Endometriosis of intestine
197033	Endometriosis of pelvic peritoneum
199881	Endometriosis of ovary
200461	Endometriosis of uterus
433527	Endometriosis (clinical)
608874	Endometriosis of left fallopian tube
608875	Endometriosis of right fallopian tube
608955	Endometriosis of left broad ligament
608956	Endometriosis of right broad ligament
608957	Endometriosis of left round ligament
608958	Endometriosis of right round ligament
4003678	Endometriosis of the spermatic cord following estrogen therapy
4019817	Endometriosis of broad ligament
4034015	Chocolate cyst of ovary
4034016	Endometriosis outside pelvis
4051345	Endometriosis of pleura
4058381	Endometriosis interna
4072148	Endometriosis of umbilicus
4127413	Endometriosis of myometrium
4132140	Endometriosis of pelvis
4146995	Cutaneous endometriosis
4176409	Endometriosis of lung
4182703	Endometriosis of round ligament
4187787	Deciduomatosis
4189364	Endometriosis of cervix
4195507	Endometriosis of uterosacral ligament
4200841	Endometriosis of rectovaginal septum
4211992	Endometriosis of vagina
4222798	Endometriosis of parametrium
4224161	Endometriosis of appendix

4260818	Endometriosis of colon
4264439	Extraovarian endometriosis
4272614	Endometriosis of the cul-de-sac
4276944	Endometriosis of bladder
4288543	External endometriosis
4307585	Endometriosis of vulva
4317964	Endometriosis of rectum
36713393	Endometriosis of small intestine
36713394	Endometriosis of thorax
36717630	Endometriosis of large intestine
37110261	Superficial endometriosis of pelvic peritoneum
37110262	Deep endometriosis
37117191	Superficial endometriosis of ovary
37119080	Peritoneal pockets and endometriosis
37209188	Bilateral endometriosis of ovaries
37209399	Endometriosis of right ovary
37209400	Endometriosis of left ovary

Table S9. SNOMED concepts used to define endometriosis patients, related to STAR Methods.

full condition set		
**cluster # (UCSF)**	**cluster # (UC-wide)**	**Jaccard index**
1	2	0.0785
2	2	0.0117
3	7	0.1309
4	6	0.0323
5	(no matching cluster)	
6	8	0.3102
7	8	0.0199
8	7	0.0287
9	2	0.0453
10	8	0.0409
11	8	0.1036
12	7	0.0382
13	6	0.1935
14	(no matching cluster)	
15	8	0.0084
16	7	0.0102
17	8	0.006
18	10	0.0968
19	7	0.0183
20	5	0.1699
21	22	0.2554
**pre-endometriosis condition set**		
**cluster # (UCSF)**	**cluster # (UC-wide)**	**Jaccard index**
1	20	0.125
2	35	0.1273
3	5	0.0823
4	6	0.1765
5	3	0.0833
6	9	0.3602
7	10	0.0462
8	5	0.012
9	9	0.0255

10	13	0.1449
11	5	0.0523
12	8	0.3371
13	9	0.008
14	5	0.0148
15	28	0.0674
16	5	0.0405
17	(no matching cluster)	
18	3	0.4
19	9	0.0037
20	9	0.0042
21	23	0.1711
22	10	0.0339
23	3	0.0455
24	10	0.0548
25	10	0.0303
26	(no matching cluster)	
27	10	0.0167
28	7	0.0645
29	(no matching cluster)	
30	4	0.25
31	(no matching cluster)	

Table S10. Jaccard similarity indices between clusters matched across the UCSF and UCHDW datasets, using both the full patient timelines and only pre-endometriosis diagnoses, related to Figures 3 and 4.
